# Morphological Transitions of Photoresponsive Vesicles from Amphiphilic Polypeptoid Copolymers for Controlled Release

**DOI:** 10.3390/polym12040798

**Published:** 2020-04-03

**Authors:** Xu Yang, Zhiwei Wang, Jing Sun

**Affiliations:** Key Laboratory of Biobased Polymer Materials, Shandong Provincial Education Department, School of Polymer Science and Engineering, Qingdao University of Science and Technology, Qingdao 266042, China

**Keywords:** photoresponsive, polypeptoid, self-assembly, drug delivery

## Abstract

Photoresponsive polymers have attracted increasing interest for a variety of applications. Here, we report a family of photoresponsive polypeptoid-based copolymer poly(ethylene glycol)-*b*-poly(N-(S-(*o*-nitrobenzyl)-thioethyl) glycine)-*co*-poly(N-(2-phenylethyl) glycine) (PEG-*b*-PNSN-*co*-PNPE) synthesized by the controlled ring-opening polymerization (ROP) technique. The key feature of the design is to incorporate both *o*-nitrobenzyl group moiety to offer the photoresponsive property and phenethyl residues to tune the structural and amphiphilic property of the system. We demonstrate that the cleavage degree of the o-nitrobenzyl group can reach to 100% upon UV-irradiation. With delicate design, a photoresponsive vesicle-to-sphere transition has been observed that facilitates the release of the encapsulants. This work provides a facile approach to prepare a type of photoresponsive polymers with tunable properties for drug delivery.

## 1. Introduction

Self-assemblies prepared from natural polymers and bioinspired synthetic polymers have been largely used for pharmaceutical applications due to their excellent biocompatibility and non-immunogenicity [1,2,3,4,5,6,7,8]. Stimuli-responsive self-assembly is of particular interest for versatile drug delivery as it can rapidly respond to the external stimuli in a controlled manner. Photoresponsive assemblies have attracted great attention in various fields due to the unique properties of their photoreactions, such as high electivity and efficiency. The *o*-nitrobenzyl group, as one of the photoliable groups, shows excellent stability in extreme conditions with tunable photochemical properties [1,9,10,11,12,13]. Photo cleavage of polymers containing *o*-nitrobenzyl group can generate amine, carboxylic acid, and thiol groups with high reactivity [2,14,15,16,17,18,19]. Consequently, the physicochemical properties of the assemblies largely vary that result in morphology transition or disassembly and a further release of the encapsulated payloads. The photo-responsive self-assemblies are considered as excellent tools for smart drug delivery. 

Polypeptoids, also known as nitrogen-substituted polyglycines (N-polyglycine), are a class of bioinspired polymer materials with biological activity. The properties of the polypeptoids are mainly determined by the side chain moiety because of the absence of hydrogen bonding and chirality in the backbone [20,21,22,23,24,25,26,27]. Both structural and property’s tunablity enables the polypeptoid–based system highly designable. We have previously prepared a type of photoresponsive polypeptoid-based diblock copolymers that can self-assemble into various morphologies. After UV-irradiation, more than one-fourth of the *o*-nitrobenzyl group and the nanostructures remain due to the cross-linking of the disulfide bonds. To benefit for the smart drug delivery application, in this study we synthesized a class of photoresponsive block copolymer poly(ethylene glycol)-*b*-poly(N-(S-(*o*-nitrobenzyl)-thioethyl) glycine)-*co*-poly(N-(2-phenylethyl) glycine) (PEG-*b*-PNSN-*co*-PNPE) by statistical copolymerization of a mixture of N-(S-(*o*-nitrobenzyl)-thioethyl) and N-phenethyl N-carboxyanhydrides (NCAs) using amino-terminated poly(ethylene glycol) (mPEG–NH_2_) as the macroinitiator by ring-opening polymerization (ROP). The advantage of the design is to incorporate both *o*-nitrobenzyl group moiety to offer the photoresponsive property and phenethyl residues to tune the structural and amphiphilic property of the system. We demonstrated that the *o*-nitrobenzyl (NB) group can be entirely cleaved to induce the free thiol groups as the irradiation time increases up to 10 h. Simultaneously, the self-assembled morphology transforms from a few hundred nanometers of vesicles to tens of nanometers of nanospheres. This transition further results in the release of the small molecule in a controlled manner. The synthesis, self-assembly and phototriggered drug release have been thoroughly investigated.

## 2. Materials and Methods

### 2.1. Materials and Instruments

Hexane, tetrahydrofuran (THF), and dichloromethane (DCM) were purified by purging with dry nitrogen, followed by passing through columns of activated alumina. *N*,*N*-Dimethylformamide (DMF) was treated with free amine scavenger (Aldrich, Berlin, Germany) before passing through 4 Å molecular sieves and activated alumina column. Ethyl acetate (EtOAc) was freshly distilled from CaH_2_. Methoxypolyethylene glycol amine (mPEG–NH_2_, Mw = 2000 g/mol) was purchased from Jenkem Technology Co, Ltd. (Beijing, China). *o*-nitrobenzyl bromide was purchased from Ark Pharm reagent Co., Ltd. (Chicago, IL, USA). Cysteamine hydrochloride (98%) was purchased from Admas Reagent Co., Ltd. (Shanghai, China). Phenylethylamine, di-tert-butyl dicarbonate, rhodamine B and glyoxylic acid monohydrate were purchased from Titan reagent (Shanghai, China). All other chemicals were purchased from commercial suppliers and used without further purification unless otherwise noted.

^1^H NMR spectra were recorded on Bruker AV400 FT-NMR spectrometer. Gel permeation chromatography/laser light scattering (GPC/LLS) was performed at 50 °C using an SSI (Series I) pump (LC20AT, Shimadzu Corporation, Kyoto, Japan) connected to Wyatt Optilab DSP with 0.02 M LiBr in DMF as the eluent at a flow rate of 1.0 mL/min. Conventional calibrations were performed using polystyrene standards (PS). The concentrations of all samples were about 5 mg/mL. Differential scanning calorimetry (DSC) studies were conducted using a TA instrument DSC 25 (New Castle, DE, USA). Powder samples enclosed in the aluminum pans were heated from −50 to 200 °C at 10 °C min^−1^ for 3 cycles. Transmission electron microscopy (TEM) samples were examined with a JEM2200FS TEM (200 keV, Tokyo, Japan). TEM samples were prepared by pipetting polymer solution on carbon coated TEM grids and the excess amount of solution was blotted with a piece of filter paper. The hydrodynamic diameter (*D*_h_) of the assemblies was recorded at 25 °C by dynamic light scattering (DLS) using a Brookhaven NanoBrook instrument (Holtsville, NY, USA). The UV–vis spectra of the samples were recorded at room temperature using a Shimadzu UV-2910 spectrometer. The sample was irradiated using a mercury high pressure UV lamp (250 W, GGZ250, Shanghai Jiguang Special Lighting Electrical Factory, Shanghai, China). The samples were stored avoiding exposure from nature light.

### 2.2. Synthesis of Poly(ethylene glycol)-b-poly(N-(S-(o-nitrobenzyl)-thioethyl) glycine)-co-poly(N-(2-phenylethyl) glycine) (PEG-b-PNSN-co-PNPE) Copolymer

The synthesis of N-(S-(*o*-nitrobenzyl)-thioethyl) and N-phenylethyl N-carboxyanhydrides monomers has been reported in previous work [28,29]. mPEG–NH_2_ was completely dried under vacuum for 30 h and dissolved in anhydrous THF in a polymerization tube. The solution of NSN–NCA and NPE–NCA mixtures (100 mg/mL in THF) at the given ratio was then added to the polymerization tube. The polymerization was performed under a N_2_ atmosphere at 55 °C and monitored by FTIR spectrum until the characteristic peaks (1850 and 1790 cm^−1^) of NCAs disappeared. The final solution was precipitated into excess cold diethyl ether. The pale yellow oil was collected by centrifugation, washed another three times by cold diethyl ether and dried under reduced pressure to yield white to pale yellow oil (56% yield). ^1^H NMR (400 MHz, DMSO) δ: 3.53–2.97 (t, 70H), 3.23 (s, 3H), 3.05–3.44 (t, 70H), 3.50 (s, 176H), 3.76–4.70 (s, 92H), 6.87–7.35 (m, 120H), 7.36–7.71 (m, 33H), and 7.85–8.05 (d, 11H).

In the entire procedure, the polymer was avoided exposure to nature light.

### 2.3. Cleavage of Copolymers with UV Irradiation

Sixty milligrams of PEG-*b*-PNSN-*co*-PNPE copolymer was dissolved in 6 mL of anhydrous DCM in a glass vial and 0.01 equivalent of dimethylphenylphosphine (DMPP) was added. Afterwards, the solution was exposed to a mercury high pressure UV lamp. 1 mL of the solution was taken at intervals of 2, 4, 6, 8, and 10 h, respectively, and precipitated with an excess of diethyl ether for three times. The product was dried under vacuum at 30 °C before measurement.

### 2.4. Preparation of Assemblies in Aqueous Solution

The PEG-*b*-PNSN-*co*-PNPE copolymer (10 mg) was first dissolved in 1 mL of THF, then 20 mL of deionized water was slowly added dropwise with stirring. After the addition of water was completed, the solution was stirred at room temperature for another 48 h. Then the THF was removed by a rotary evaporator. The solution was stirred under a mercury high pressure UV lamp for 10 h.

### 2.5. Preparation of Rhodamine B Loaded Vesicles in Aqueous Solution

Ten milligrams of PEG-*b*-PNSN-*co*-PNPE was dissolved in 1 mL of THF, and then 5 mg of rhodamine B in aqueous solution (20 mL) was slowly dropped into the polymer solution with stirring. The solution was stirred for another 48 h at room temperature and then dialyzed (MWCO = 3500, molecular weight cutoff) against deionized water to remove unloaded rhodamine B. The solution outside the dialysis bag was measured by a UV-vis spectrometer at 553 nm and the weight of the loaded drug was then obtained.

### 2.6. In Vitro Drug Release Profiles

Five milliliters of the rhodamine B-loaded vesicles solution (0.5 mg/mL) was placed in a dialysis bag (MWCO = 3500) and dialyzed against 25 mL of PBS buffer at 37 °C with continuous shaking. The dialysis solution was periodically replaced, and the value at 553 nm was recorded by a UV-vis spectrometer and the weight of the released drug was calculated. For the UV-irradiated sample, the same procedure was performed except that the solution was first irradiated under the UV lamp for 6 h.

## 3. Results and Discussion

**Scheme 1:**
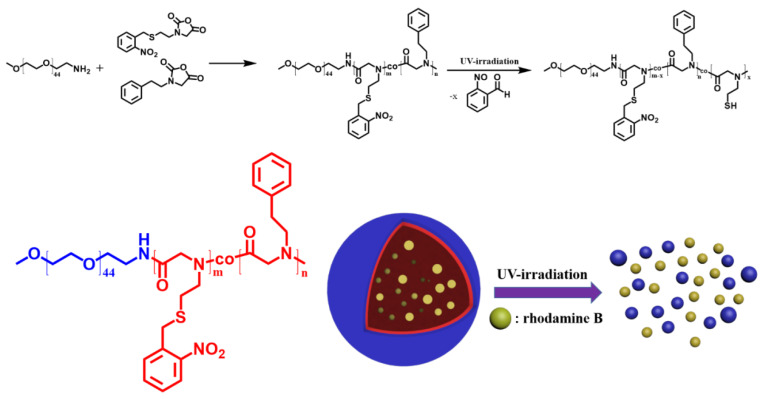
The phototriggered assemblies from the poly(ethylene glycol)-*b*-poly(N-(S-(*o*-nitrobenzyl)-thioethyl) glycine)-*co*-poly(N-(2-phenylethyl) glycine) (PEG-*b*-PNSN-*co*-PNPE) triblock random copolymer.

**Figure 1:**
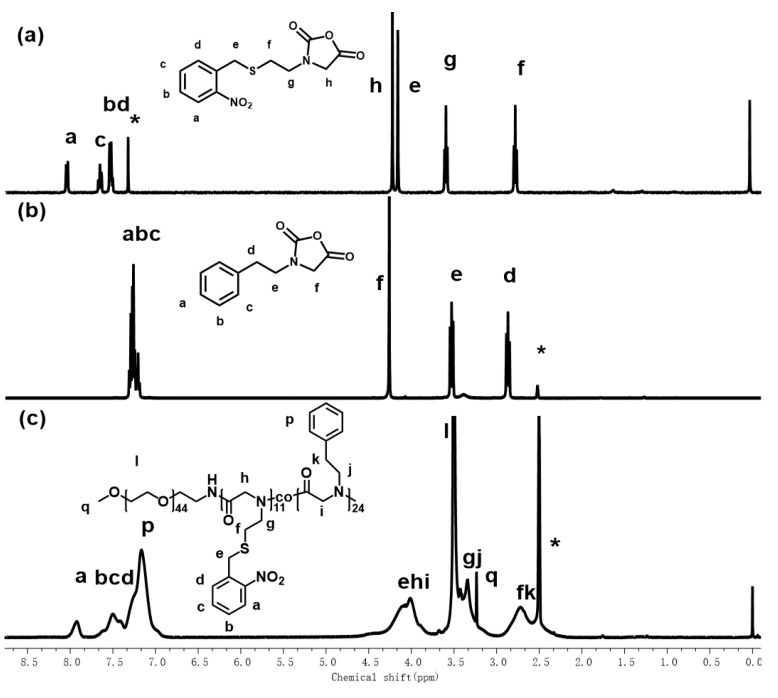
^1^H NMR spectra of (**a**) NSN–NCA in CDCl_3_, (**b**) NPE–NCA and (**c**) PEG-*b*-PNSN-*co*-PNPE in DMSO. * indicates solvents.

The N-(S-(*o*-nitrobenzyl)-thioethyl)-N-carboxyanhydride (NSN–NCA) and N-phenylethyl N-carboxyanhydride (NPE–NCA) monomers were synthesized as reported elsewhere (Scheme S1). The chemical structure of the monomers was confirmed by ^1^H NMR spectroscopy (Figure 1a,b, Figures S1 and S2). The copolymers (PEG-*b*-PNSN-*co*-PNPE) were then synthesized by statistical copolymerization of a mixture of NSN–NCA and NPE–NCA using mPEG–NH_2_ (M_n_ = 2000 g/mol) as the macroinitiator (Scheme 1). The polymerization process was monitored by FTIR. The disappearance of two characteristic ν_C=O_ peaks of the monomer at 1780 cm^−1^ and 1850 cm^−1^ was considered as complete conversion of NCA monomers to polypeptoids. The ^1^H NMR spectra show that all peaks of the synthesized copolymers are well assigned, confirming their chemical structures (Figure 1c and Figure S3). A series of copolymers were synthesized by varying the ratio of NSN–NCA to NPE–NCA and monomers to PEG-NH_2_ macroinitiator. The average DP (degree of polymerization) of PNSN was held fixed at approximately 10 and 19, and the average DP of PNPE was varied from 10 to 34. All the molecular characteristics of the copolymers PEG-*b*-PNSN_m_-*co*-PNPE_n_ are shown in Table 1. The GPC traces (Figure S4) show unimodal peaks and narrow molecular weight distribution with dispersities (*Đ*) < 1.25 for all the diblock copolymers (Table 1). This indicates that the statistical copolymerization of PNSN and PNPE monomers is well-controlled. We investigated the thermal properties of the samples by DSC (Figure S5a). The samples with and without irradiation show the absence of crystallization and melting peaks in the entire experimental window by DSC (Figure S5). As previously reported [28], the presence of the PNSN block significantly inhibits the crystallization properties of the PEG block and the PNPE block.

**Figure 2:**
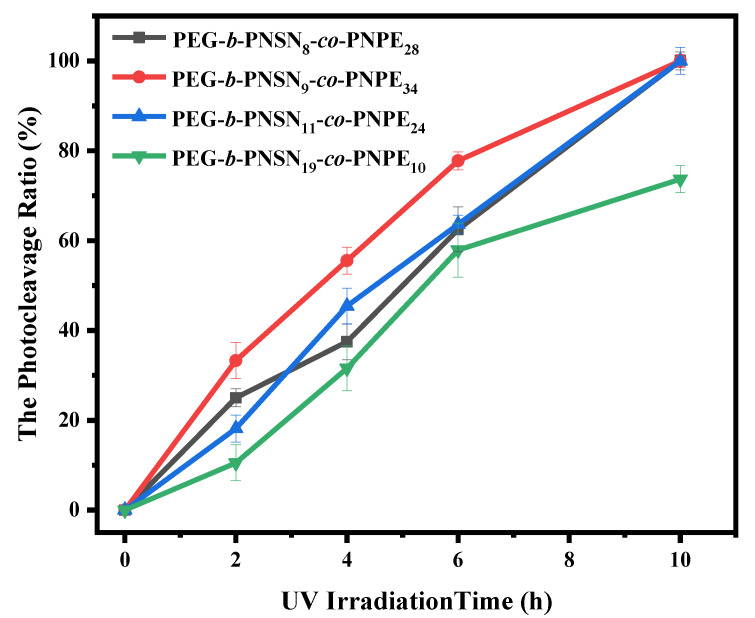
The photocleavage ratio of copolymers with different UV-irradiation time.

As expected, the obtained copolymers exhibit photoresponsive behavior upon UV irradiation. This is due to the presence of the *o*-nitrobenzyl (NB) group, which can be photocleaved to generate free thiol groups. The chemical structures of copolymers with different irradiation times were studied by ^1^H NMR, as shown in Figure S6. The NB group is gradually eliminated from the polymers during the irradiation process, as indicated by the decreased peaks at 7.4 and 7.9 ppm. Unlike previous reports, the samples (with DP_PNSN_ of ~10) remain good solubility after prolonged irradiation. This is possibly because the presence of the PNPE block increases the solubility of the system. More importantly, the PNPE may also behave as steric hindrance to reduce the number of cross-links that benefits the deprotection procedure. The ^1^H NMR results show that 100% NB groups are cleaved from the PEG-*b*-PNSN_8_-*co*-PNPE_28_, PEG-*b*-PNSN_9_-*co*-PNPE_34_, and PEG-*b*-PNSN_11_-*co*-PNPE_24_ copolymer after 10 h UV irradiation (Figure 2). In the case of PEG-*b*-PNSN_19_-*co*-PNPE_10_, merely 70% NB groups are cleaved, because of the high molar ratio of PNSN that decreases the solubility of the system.

**Figure 3:**
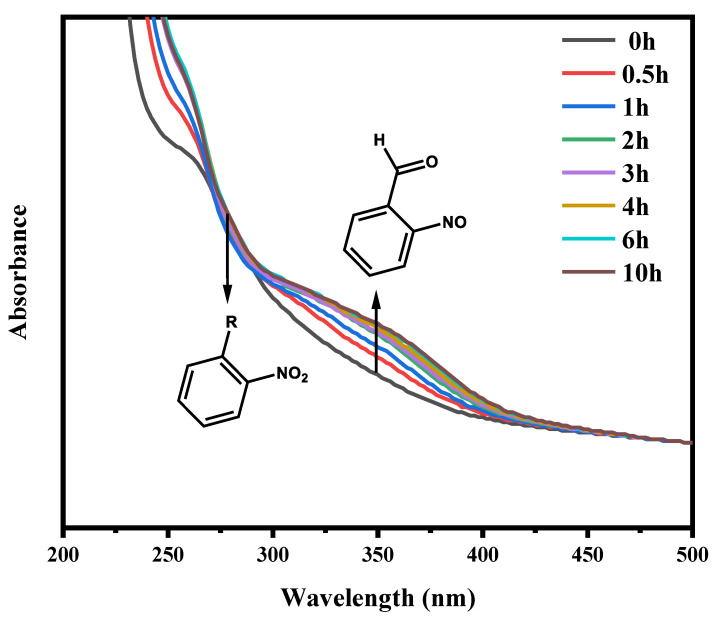
UV-vis spectra of PEG-*b*-PNSN_11_-*co*-PNPE_24_ in aqueous solution (0.5 mg/mL) with different UV-irradiation times.

We further investigated the photoresponsive properties of PEG-*b*-PNSN-*co*-PNPE assemblies in aqueous solution. The copolymer was first dissolved in THF and then deionized water was added to induce molecular assembly. After removal of THF, the final concentration of all four samples was fixed at 0.5 mg/mL. After exposure to UV light, the colorless assembled solution changed to yellow, which indicates that a photocleavage reaction occurs. This process is further monitored by UV-Vis spectrometer (Figure 3). With increasing the irradiation time, the enhanced characteristic absorption peak at 350 nm and the decreased peak at 280 nm indicate that the NB group is gradually photocleaved from the copolymer and produces 2-nitrosobenzaldehyde. This is coincident with previously reported results. The UV irradiation removes the hydrophobic NB group and free thiol group is generated. The thiol group can be further oxidized to a disulfide bond in solution, which promotes cross-linking of the components.

**Figure 4:**
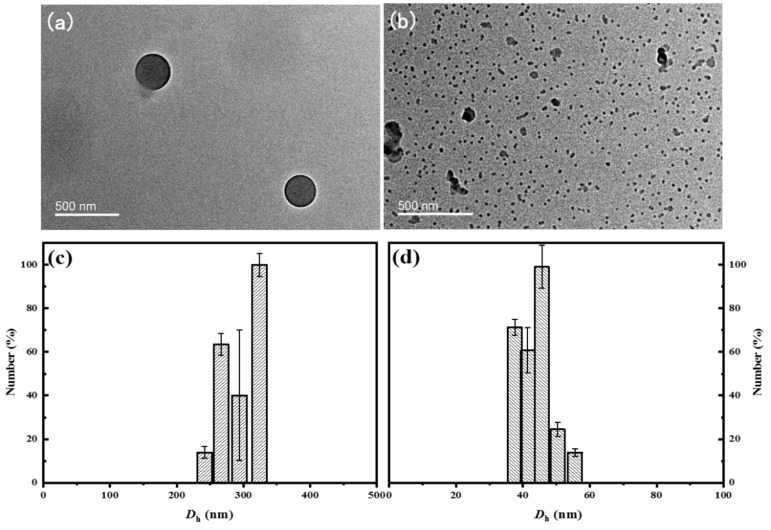
The Transmission electron microscopy (TEM) images of PEG-*b*-PNSN_8_-*co*-PNPE_28_ with non-irradiation (**a**) and with 10 h irradiation (**b**); the *D*_h_ with non-irradiation (**c**), and with 10 h irradiation (**d**) in aqueous solution.

**Figure 5:**
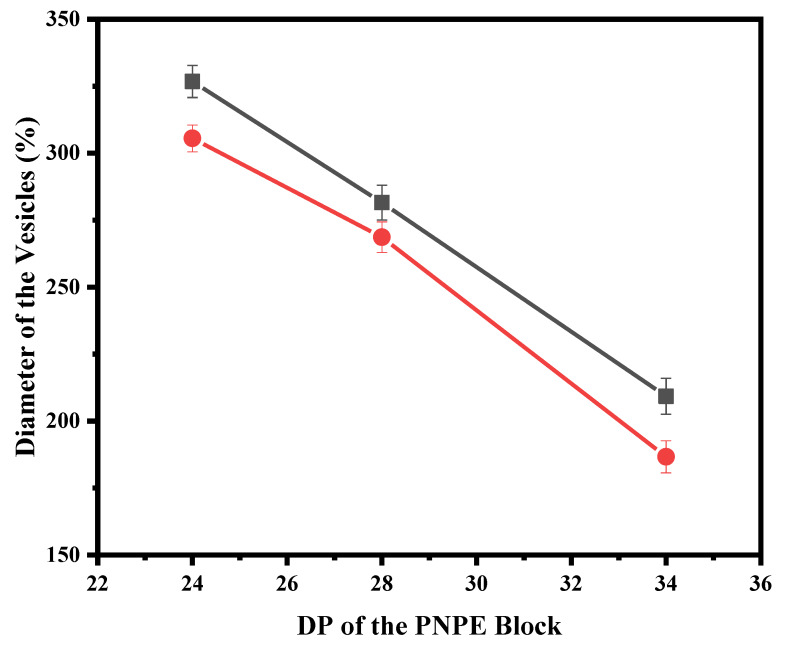
The relationship between DP(degree of polymerization) of the PNPE block and diameter of the vesicles as the DP of PNSN block is fixed at around 10. The red circle represents the results observed by TEM and the black square represents the results determined by dynamic light scattering (DLS).

To probe the nanostructure and size of the copolymer before and after UV irradiation, TEM and DLS were performed. In all cases, the samples exhibit similar self-assembly behavior (Figure 4, Figures S7 and S8). The PEG-*b*-PNSN_8_-*co*-PNPE_28_ without UV irradiation self-assembles into the vesicles with a diameter of 268.65 ± 11.4 nm, as observed by TEM (Figure 4a). The contrast between the bright center and the dark periphery suggests a vesicular hollow structure. The DLS result (Figure 4c) shows a consistent hydrodynamic diameter (*D*_h_) of 281.57 ± 10.6 nm, confirming the TEM images (Figure 4a). Note that water is a good solvent for PEG and non-solvent for hydrophobic PNSN and PNPE blocks. It is expected that the random PNSN-*co*-PNPE block forms the inner layer of the vesicle flanked by PEG out layers. As the DP of PNSN block is fixed at around 10, the size of the vesicles slightly decreases with the DP of PNPE block increasing, as indicated by the TEM images and DLS results (Figure 5). We assume that this is possibly because the increased hydrophobicity results in a reduced aggregation number for more stable nanostructures. After 10 h irradiation, the self-assembled morphology transforms into the spherical morphology with a diameter of 26.5 ± 5.1 nm by TEM (Figure 4b). Upon exposure, the NB group is cleaved, which induces the free thiol groups. The hydrophobicity the copolymer decreases significantly, which enables the vesicle-to sphere transition. Note that the thiol groups can be further oxidized into disulfide bonds in solution in the presence of oxygen. This is very different from the results of PEG-*b*-PNSN in a previous report, where the morphology is stabilized by the disulfide bonds [28]. In this case, the random distribution of PNSN and PNPE blocks may disrupt the cross-linking of thiol groups on PNSN. Thus, the morphology transition occurs prior to cross-linking. The *D*_h_ of the spheres measured by DLS (Figure 4d) is 43.4 ± 8.6 nm, larger than that observed from TEM measurement (Figure 4b). This may be due to the volume shrinkage of the cross-linked assemblies during drying process and invisible corona with low electron density by TEM [3]. All the other copolymers show similar behavior (Figure S7). With fixed DP of PNSN, the size of the micelles decreases with the DP of PNPE block increasing, similar to that before UV-irradiation (Figure 5).

**Figure 6:**
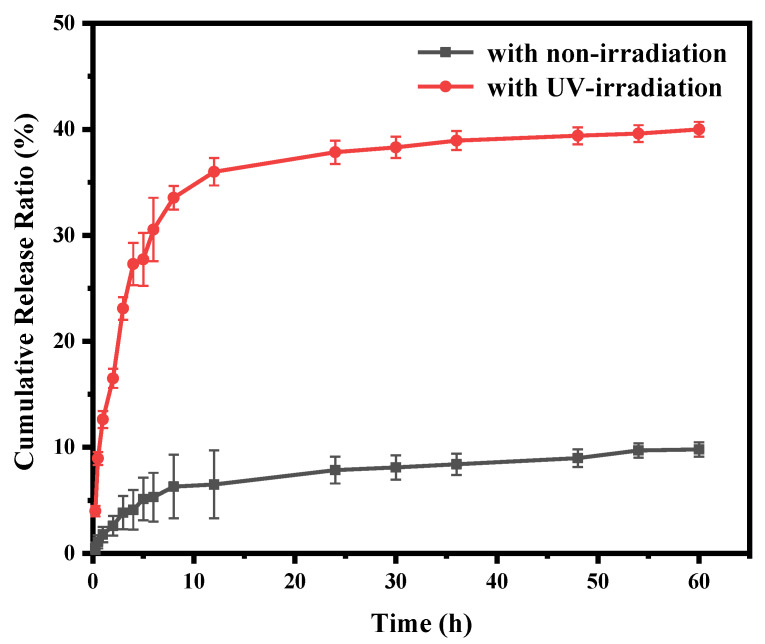
In vitro drug-release profiles of rhodamine B loaded PEG-*b*-PNSN_19_-*co*-PNPE_10_.

The dramatic morphology variation facilitates the prompt release of encapsulates. Thus, we designed and studied the drug loading and release capacity of the PEG-*b*-PNSN-*co*-PNPE carrier. Note that most of the studies focus on the encapsulation of the hydrophobic drugs such as doxorubicin [3]. Considering the aqueous interior of the vesicle, we used a hydrophilic fluorescent probe rhodamine B for further study. Rhodamine B was loaded into the PEG-*b*-PNSN_19_-*co*-PNPE_10_ vesicles by co-solvent assembly and subsequent dialysis, which results in the load capacity of 34.5 wt.% by UV-Vis spectrometer. The assembled solutions with/without irradiation were then placed in PBS (37 °C) simultaneously for further investigation of in vitro release profile. Figure 6 shows that both systems show similar release kinetics, where rapid release is observed in the initial stage and turns to modest increase tendency. Further, the sample after 6 h irradiation shows much pronounced release rate compared with the non-irradiated sample (Figure 6). Upon irradiation, the cumulative drug release reached ~40% in 60 h, which is nearly four-times than that of non-irradiated samples. This variation suggests that the hydrophilic molecules diffuse dramatically from the vesicles due to the photo-triggered morphology transition. Our system provides a new solution for the photo-responsive delivery of hydrophilic drugs for biomedical applications.

## 4. Conclusions

In summary, we have successfully synthesized a family of photoresponsive polypeptoid-based copolymer poly(ethylene glycol)-*b*-poly(N-(S-(*o*-nitrobenzyl)-thioethyl) glycine)-*co*-poly(N-(2-phenylethyl) glycine) (PEG-*b*-PNSN-*co*-PNPE) by controlled ring-opening polymerization (ROP). Both *o*-nitrobenzyl group and phenethyl residues have been simultaneously incorporated to tune the property of the system. With UV-irradiation the free thiol groups can be generated by photocleavage of the *o*-nitrobenzyl (NB) groups. The self-assembled morphology transforms from vesicles of a few hundred nanometers to nanospheres of tens nanometers. This process further results in the controllable release of the small molecule by varying the UV-irradiation time. This work provides a facile approach to prepare a series of photoresponsive drug carriers with tunable properties.

## Figures and Tables

**Table 1:** Characteristics of the triblock random copolymers synthesized.

Samples	Feed Ratio ^a^	M_n_^b^ (kDa)	M_n_^c^ (kDa)	Dispersity (*Đ*) ^c^
PEG-*b*-PNSN_8_-*co*-PNPE_28_	1/10/30	8524	9833	1.25
PEG-*b*-PNSN_9_-*co*-PNPE_32_	1/10/40	9420	12,500	1.17
PEG-*b*-PNSN_11_-*co*-PNPE_24_	1/10/20	8636	11,410	1.14
PEG-*b*-PNSN_19_-*co*-PNPE_10_	1/20/10	8398	9393	1.18

^a^ Feed molar ratio of PEG/NSN–NCA/NPE–NCA. ^b^ Calculated from ^1^H NMR spectra. ^c^ Determined from Gel permeation chromatography (GPC).

## Supplementary Materials

The following are available online at https://www.mdpi.com/2073-4360/12/4/798/s1. Scheme S1: Synthetic route of NSN–NCA and NPE–NCA monomer; Figures S1–S3: ^1^H NMR spectra of NSN–NCA, NPE–NCA, and PEG-*b*-PNSN_m_-*co*-PNPE_n_; Figure S4: GPC traces of the triblock random copolymers; Figure S5: DSC thermograms of PEG-*b*-PNSN_9_-*co*-PNPE_34_ with non-irradiation (a) and with 10 h irradiation (b); Figure S6: ^1^H NMR spectra PEG-*b*-PNSN_m_-*co*-PNPE_n_ with different UV-irradiation time; Figure S7: The TEM images of PEG-*b*-PNSN_m_-*co*-PNPE_n_ with non-irradiation and with 10 h irradiation in aqueous solution; Figure S8: The diameter of PEG-*b*-PNSN_m_-*co*-PNPE_n_ with non-irradiation and with 10 h irradiation in aqueous solution determined by DLS.
