# Supplementary material for: Morphological Transitions of Photoresponsive Vesicles from Amphiphilic Polypeptoid Copolymers for Controlled Release
Source: Polymers (Basel). 2020 Apr 3;12(4):798. doi: 10.3390/polym12040798 (PMC7240382; doi:10.3390/polym12040798)
Supplement: Supplementary file 1 [file polymers-12-00798-s001.pdf]

Supplementary Materials: Morphological Transitions of Photoresponsive Vesicles from Amphiphilic Polypeptoid Copolymers for Controlled Release

Xu Yang, Zhiwei Wang, Jing Sun*

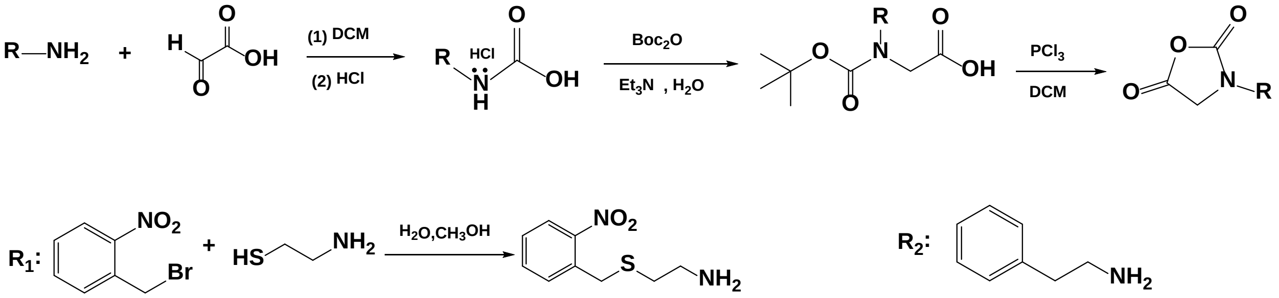

Scheme S1. Synthetic route of NSN-NCA and NPE-NCA monomer.

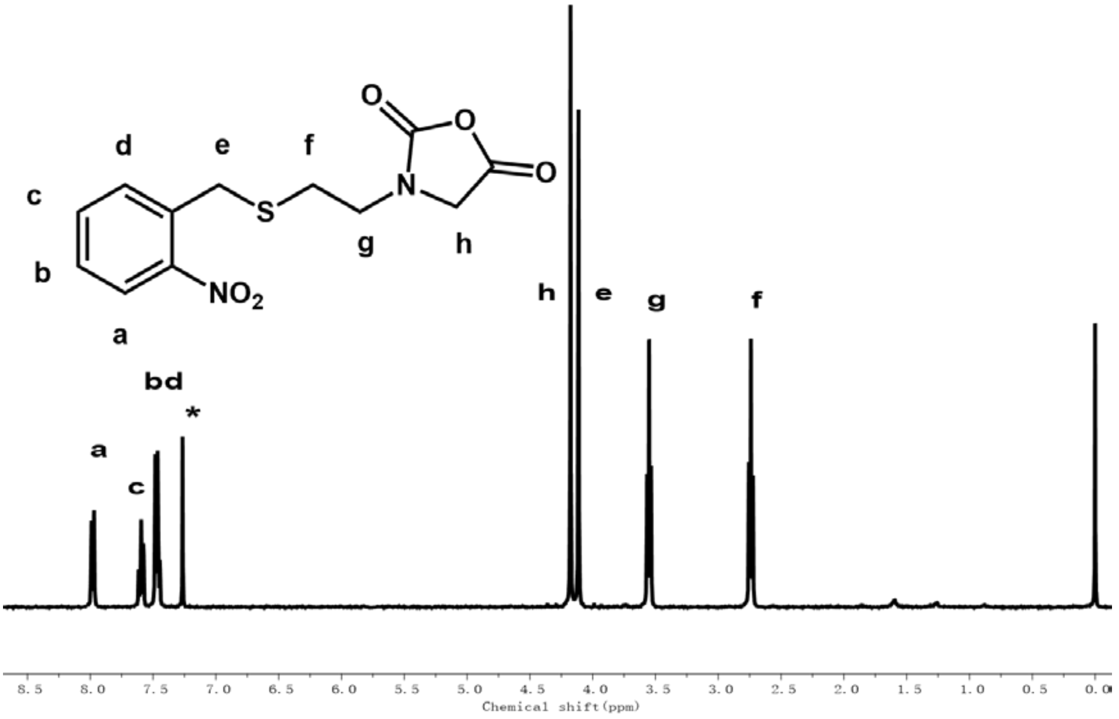

Figure S1. ^{1}H NMR spectra of NSN-NCA ($CDCl_3$, δ, ppm): 2.74 (t, 2H), 3.55 (t, 2H), 4.11 (s, 2H), 4.17 (s, 2H), 7.43-7.50 (m, 2H), 7.59 (t, 1H), 7.97 (d, 1H). * indicates solvents.

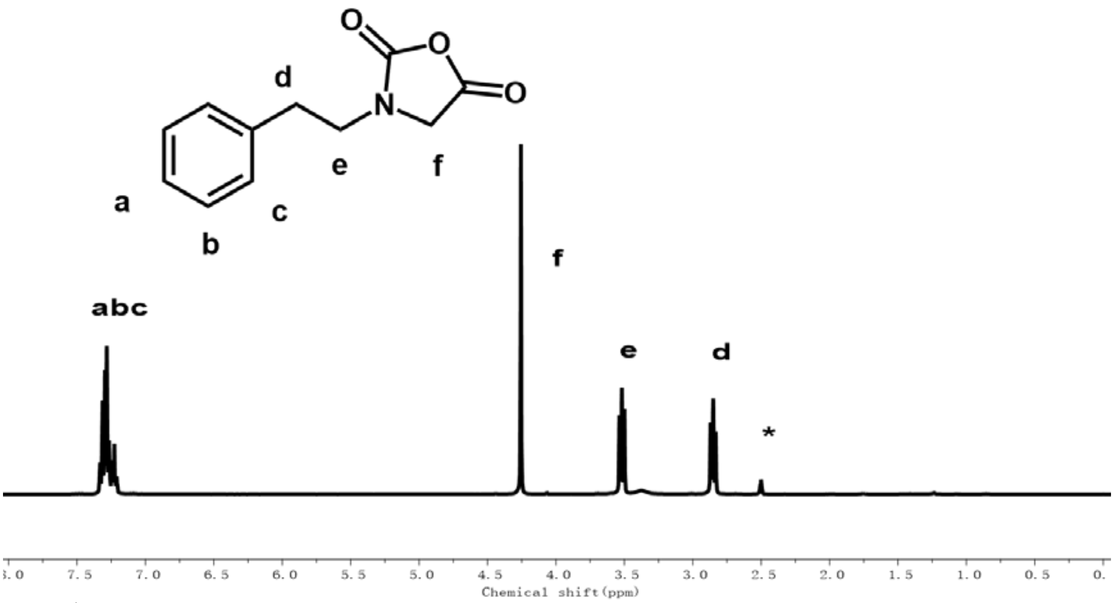

Figure S2. ^{1}H NMR spectra of NPE-NCA (DMSO, δ, ppm): 2.85 (t, 2H), 3.51 (t, 2H), 4.25 (s, 2H), 7.19-7.35 (m, 5H). * indicates solvents.

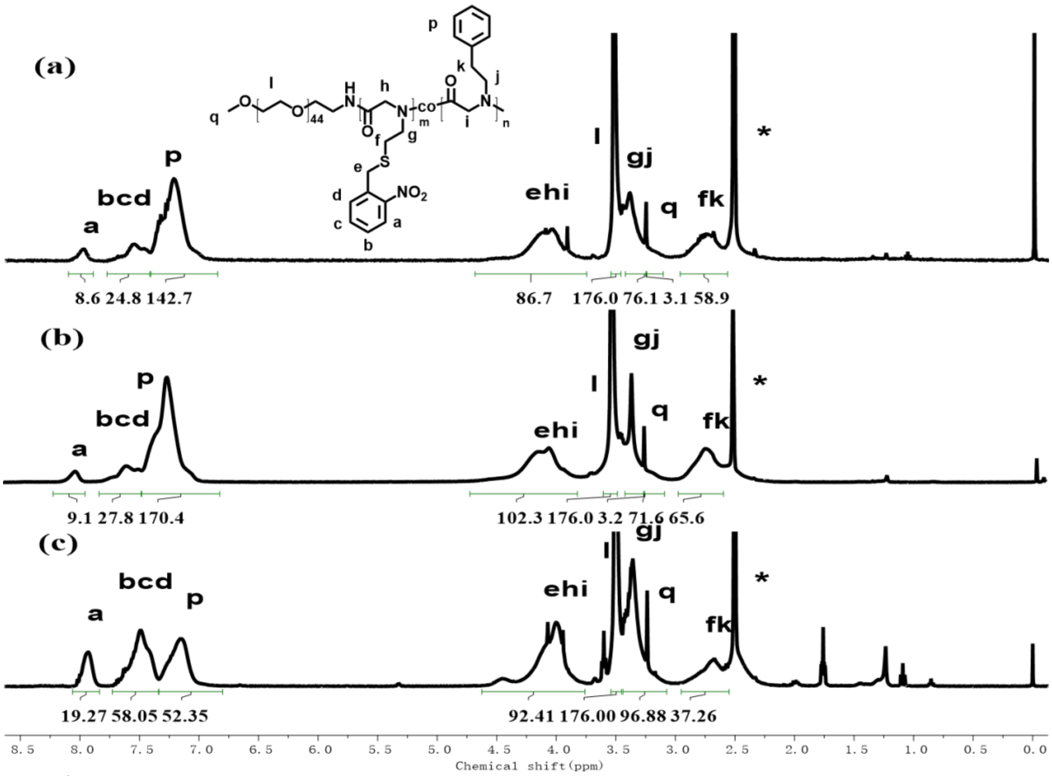

Figure S3. ^{1}H spectra of (a) PEG-*b*-PNSN$_8$-*co*-PNPE$_{28}$, (b) PEG-*b*-PNSN$_9$-*co*-PNPE$_{34}$, (c) PEG-*b*-PNSN$_{19}$-*co*-PNPE$_{10}$ in DMSO. * indicates solvents.

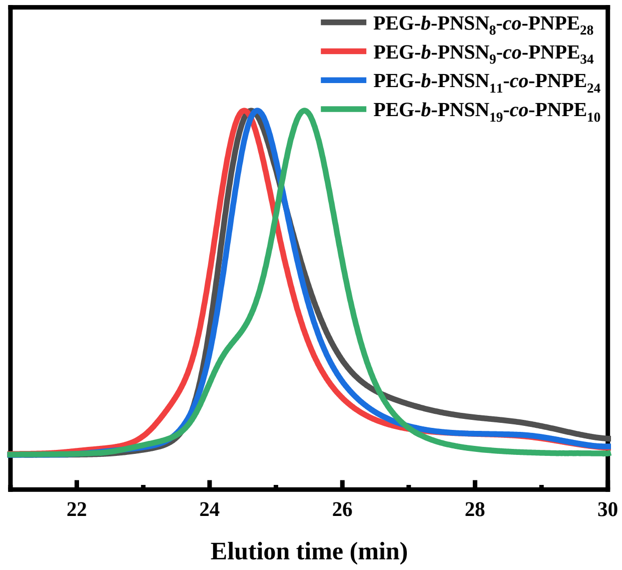

Figure S4. GPC traces of the triblock random copolymers.

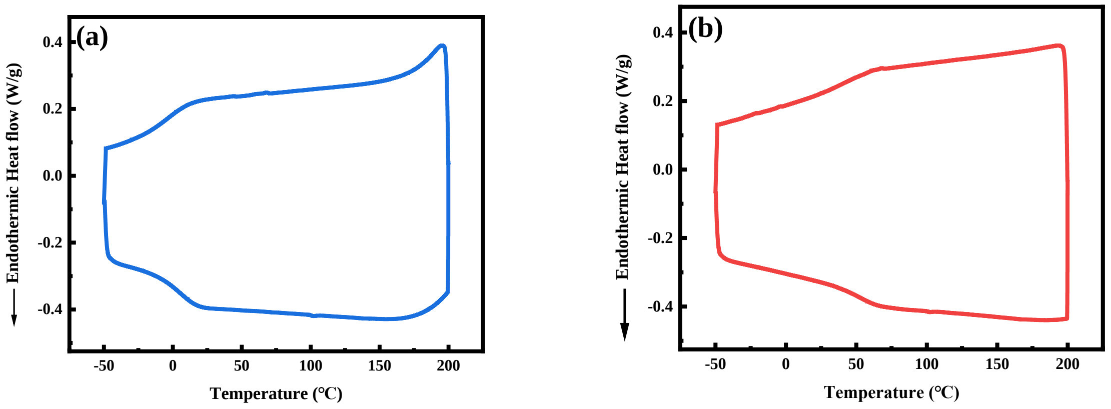

Figure S5. DSC thermograms of PEG-*b*-PNSN$_9$-*co*-PNPE$_{34}$ with non-irradiation (a) and with 10 h irradiation (b).

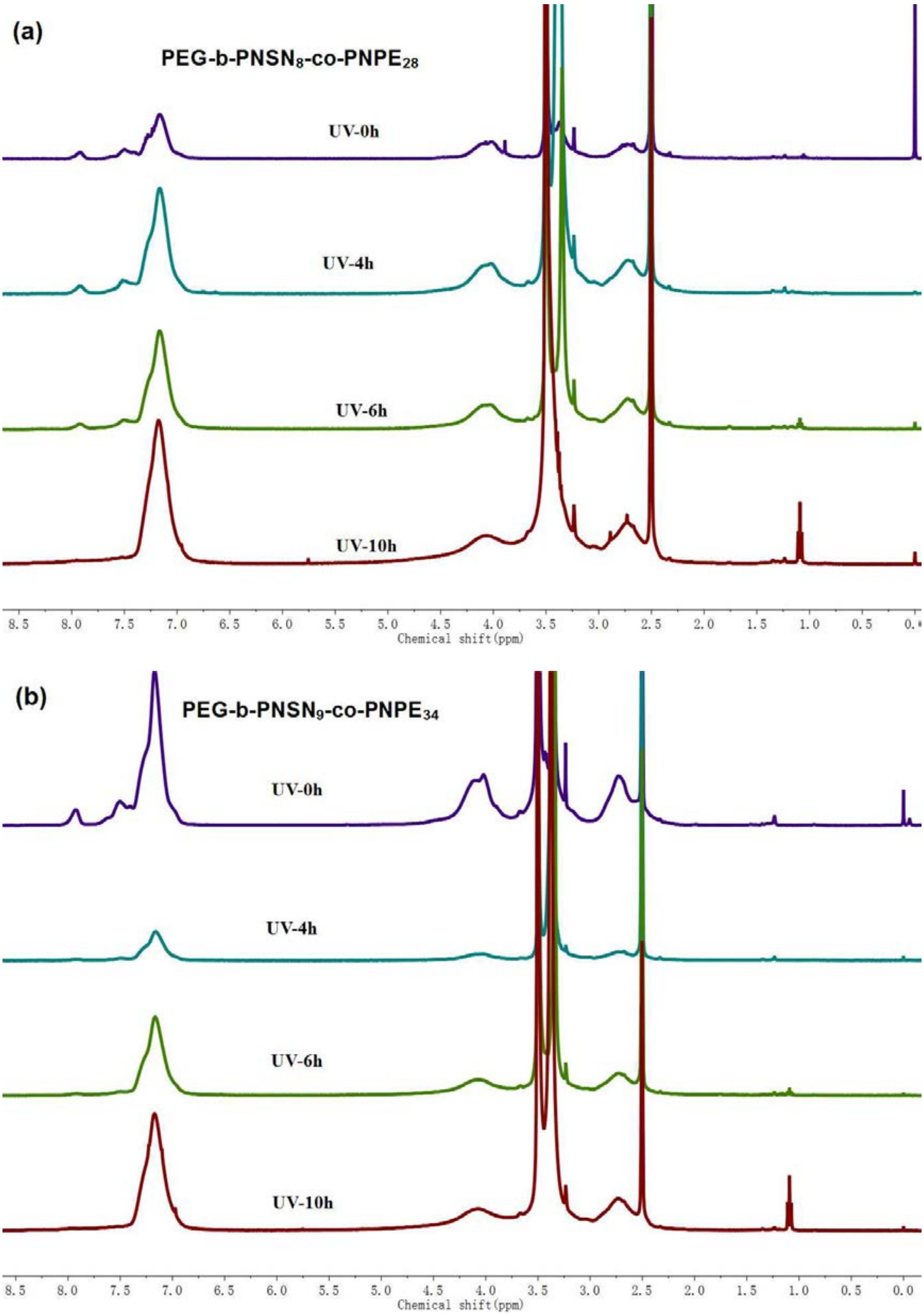
(a)
PEG-b-PNSN8-co-PNPE28
UV-0h
UV-4h
UV-6h
UV-10h
8.5 8.0 7.5 7.0 6.5 6.0 5.5 5.0 4.5 4.0 3.5 3.0 2.5 2.0 1.5 1.0 0.5
Chemical shift(ppm)
(b)
PEG-b-PNSN9-co-PNPE34
UV-0h
UV-4h
UV-6h
UV-10h
8.5 8.0 7.5 7.0 6.5 6.0 5.5 5.0 4.5 4.0 3.5 3.0 2.5 2.0 1.5 1.0 0.5 0.0
Chemical shift(ppm)

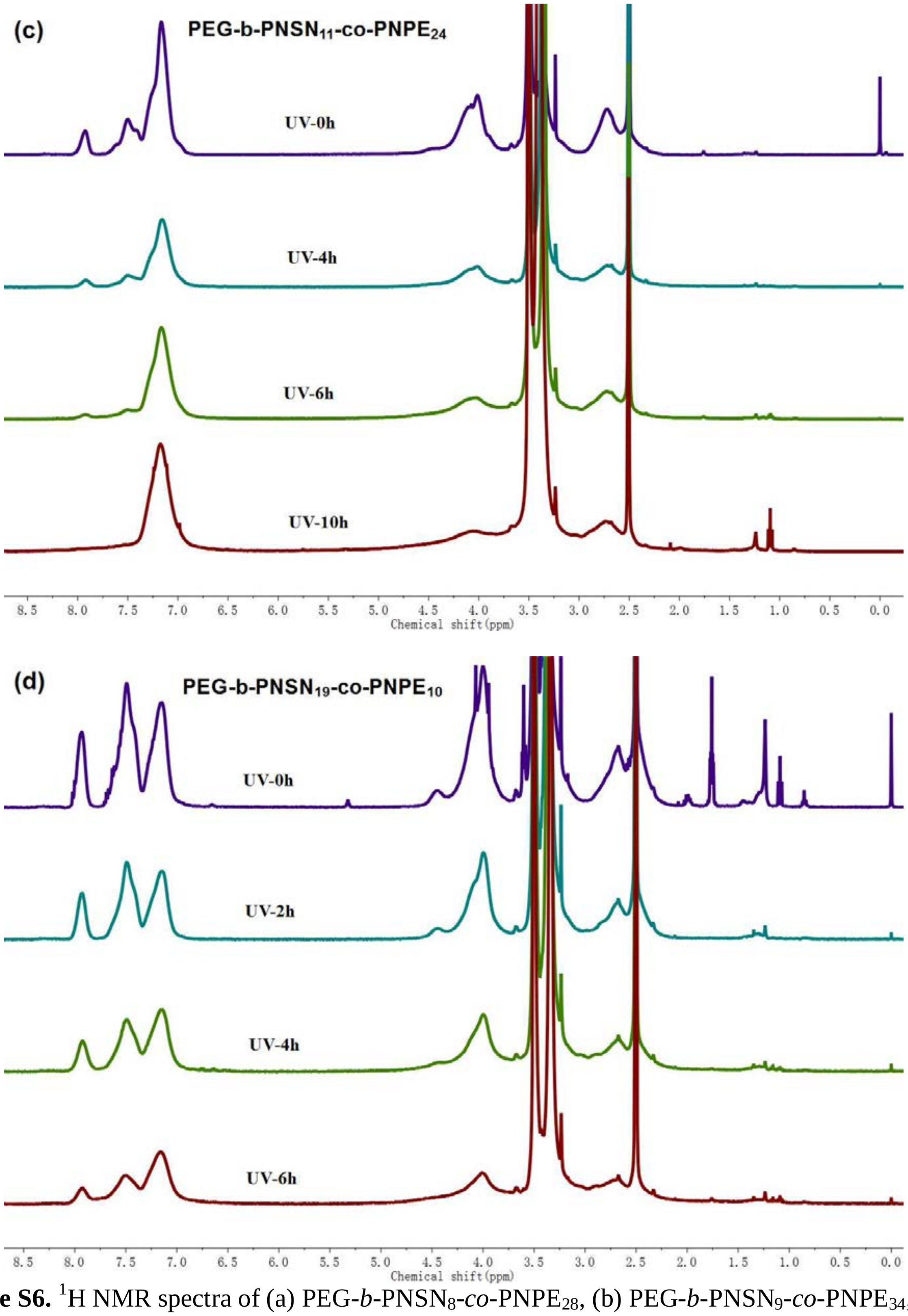

Figure S6. ^{1}H NMR spectra of (a) PEG-*b*-$PNSN_8$-*co*-$PNPE_{28}$, (b) PEG-*b*-$PNSN_9$-*co*-$PNPE_{34}$, (c) PEG-*b*-$PNSN_{11}$-*co*-$PNPE_{24}$ and (d) PEG-*b*-$PNSN_{19}$-*co*-$PNPE_{10}$ with different UV-irradiation time.

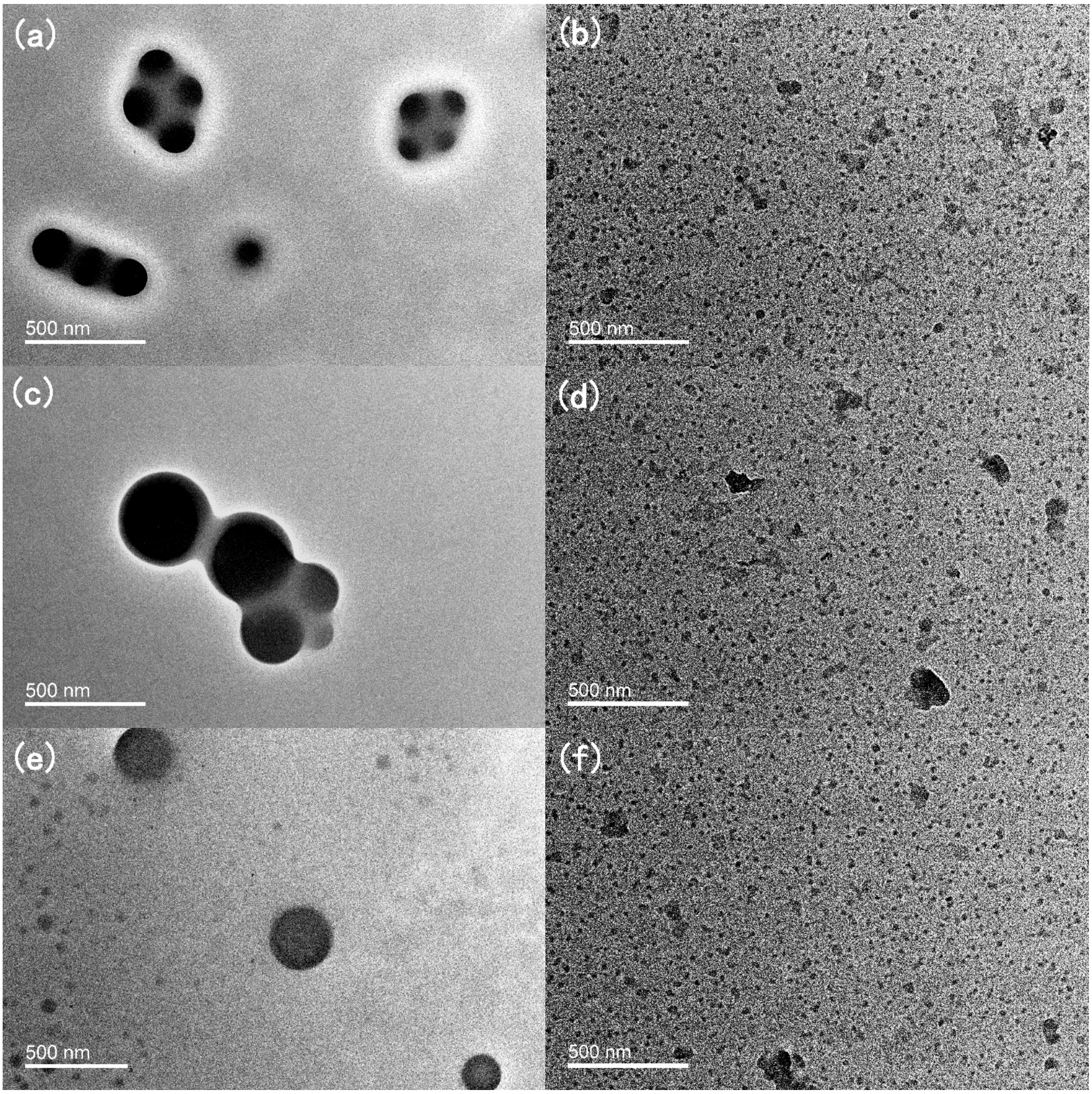

Figure S7. TEM images of PEG-*b*-$PNSN_9$-*co*-$PNPE_{34}$ with non-irradiation (a) and with 10h irradiation (b); PEG-*b*-$PNSN_{11}$-*co*-$PNPE_{24}$ with non-irradiation (c) and with 10 h irradiation (d); PEG-*b*-$PNSN_{19}$-*co*-$PNPE_{10}$ with non-irradiation (e) and with 10 h irradiation (f) in aqueous solution.

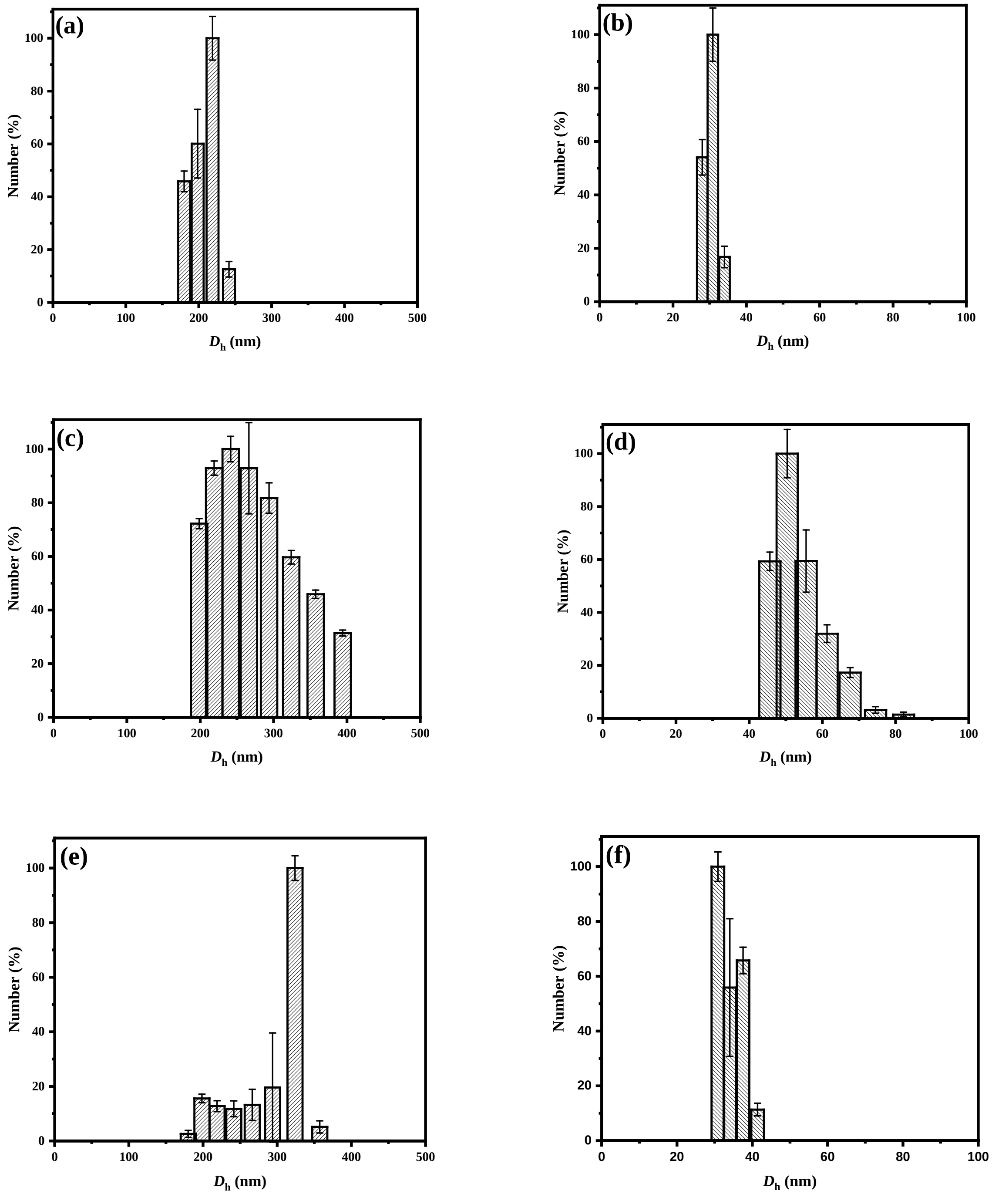

Figure S8. The D_h of PEG-*b*-$PNSN_9$-*co*-$PNPE_{34}$ with non-irradiation (a) and with 10h irradiation (b); PEG-*b*-$PNSN_{11}$-*co*-$PNPE_{24}$ with non-irradiation (c) and with 10 h irradiation (d); PEG-*b*-$PNSN_{19}$-*co*-$PNPE_{10}$ with non-irradiation (e) and with 10 h irradiation (f) in aqueous solution determined by DLS.
